# Performances of a portable electrospinning apparatus

**DOI:** 10.1007/s10529-014-1760-6

**Published:** 2015-01-01

**Authors:** Pierre-Alexis Mouthuy, Lukasz Groszkowski, Hua Ye

**Affiliations:** 1 Nuffield Department of Orthopaedics, Rheumatology and Musculoskeletal Sciences, Botnar Institute of Musculoskeletal Sciences, University of Oxford, Oxford, OX3 7LD UK; 2 Department of Engineering Science, Institute of Biomedical Engineering, University of Oxford, Old Road Campus, Oxford, OX3 7DQ UK

**Keywords:** Electrospinning, Electrospraying, Nanofibres, Portable device, Tissue engineering scaffolds, Wound dressings, Wound healing

## Abstract

To demonstrate that portable electrospinning devices can spin a wide range of polymers into submicron fibres and provide a mesh quality comparable to those produced with benchtop machines. We have designed a small, battery-operated electrospinning apparatus which enables control over the voltage and the flow rate of the polymer solution via a microcontroller. It can be used to electrospin a range of commonly used polymers including poly(ε-caprolactone), poly(p-dioxanone), poly(lactic-co-glycolic acid), poly(3-hydroxybutyrate), poly(ethylene oxide), poly(vinyl acohol) and poly(vinyl butyral). Moreover, electrospun meshes are produced with a quality comparable to a benchtop machine. We also show that the portable apparatus is able to electrospray beads and microparticles. Finally, we highlight the potential of the device for wound healing applications by demonstrating the possibility of electrospinning onto pig and human skins. Portable electrospinning devices are still at an early stage of development but they could soon become an attractive alternative to benchtop machines, in particular for uses that require mobility and a higher degree of flexibility, such as for wound healing applications.

## Introduction

Electrospinning is currently one of the most successful methods used to produce polymer submicron fibres. Compared to other techniques, it has the advantages of being simple, versatile, and cost effective. Similarly to electrospraying, which produces particles, electrospinning uses high voltage electrical charges to draw nanoscale and microscale fibres from a polymer solution (Reneker and Yarin 2008).

The submicron scale dimensions of the fibres and the high surface area to volume ratio of the meshes provide electrospun materials with unique properties. So far, they have found applications in many areas including aerospace and aviation, automotive, electronics and semiconductors, energy, filtration, textiles, cosmetics, and medicine. In the medical area, electrospun fibres can be used as scaffolds for tissue engineering, wound healing devices and drug-delivery systems. Their potential as scaffolds for tissue engineering applications mainly relies on their ability to mimic the extracellular matrix surrounding cells in tissues and organs. In particular, they have been shown to improve cell adhesion, proliferation and differentiation (Rho et al. 2006; Zonari et al. 2012; Downing et al. 2013). Promising results have been reported for their use in the repair of various biological tissues including skin, tendon, ligament, bone and cartilage (Kumbar et al. 2008; Sahoo et al. 2006; Aviss et al. 2010; Mouthuy et al. 2010; Alves da Silva et al. 2010). As a wound dressing, the high porosity, small pore size and high specific surface area of the meshes provide a physical barrier against germs and are favourable to exude fluid from the wound. Meshes can be used to prevent postsurgery adhesions (Zong et al. 2004). Therapeutic and antimicrobial agents can be incorporated into the fibres to improve the quality of the healing and minimise the risk of infection (Zamani et al. 2013; Yang et al. 2012; Zong et al. 2004). Another approach proposed to assist the healing process is to electrospun living cells together with the fibres (Townsend-Nicholson and Jayasinghe 2006; Jayasinghe 2013).

A typical electrospinning setup consists of a high voltage power supply unit, a syringe pump unit and a set of electrodes. Although not necessary for the process itself, syringe pumps are crucial to provide high quality and reproducible samples. Over the last decade, the development of electrospinning equipment for research use has been driven by the need for better control over the parameters involved to lead to better reproducibility. For industrial applications, the main focus has been on scaling up the technology to increase the rate of production. Here, we believe that remodelling the cumbersome apparatus into a small device could have advantages in terms of transportability, flexibility of use and cost. For medical applications, the ability to spray or spin directly onto a wound with a small device could allow new ways to improve tissue healing and reduce scar formation.

Several attempts have been made to miniaturise the electrospinning technology into small portable devices. However, patents have been a major brake to the dissemination of these devices as they have remained unexploited. A series of portable electrodispersing devices which could be handheld, battery-powered and offering controlled fluid delivery with piezoelectric diaphragm pumps were developed in Oxford by Pirrie and Coffee (Coffee 1998; Pirrie and Coffee 2003). Although these were aimed at electrospraying applications, the authors briefly demonstrated the potential for producing fibres. Devices dedicated to electrospinning were later developed by Reneker’s group at the University of Akron, which proposed a battery-powered device with a reservoir to provide the fluid (Smith et al. 2004). Shortly after, in 2006, Greiner and collaborators from the University of Marburg disclosed a handheld electrospinning equipment with which they performed demonstrations (Greiner and Wendorff 2007). The high voltage was supplied by standard batteries and the device had a modular construction to enable the spinning of different polymer solutions which were provided from vials. A device based on a similar principle has later been proposed by Ramakrishna’s group at the University of Singapore (Liu et al. 2010). More recently, a simple device involving a finger-pressed syringe was developed at the University of Qingdao (Long et al. 2012). However, for all these devices developed, very little technical details were released and there has been no follow up on the potential applications or actual uses. Moreover, apart from the electrospraying devices, none of the apparatus mentioned above offers a controlled fluid delivery by electronic components.

Here, we propose a novel portable electrospinning apparatus. Both the voltage and flow rate can be adjusted via a microcontroller over a range of values suitable for most electrospinning and electrospraying applications. We show its ability to produce submicron fibres and particles made of several polymers commonly used in the field. We also compare the performance of the device to a typical benchtop setup. Finally, we demonstrate the possibility of electrospinning directly onto skin to highlight the potential of the apparatus for wound healing applications.

## Materials and methods

### Electrospinner: components and manufacture

To provide the high voltage, a converter model F101 (EMCO High Voltage Corporation, US) was selected. A T-NA linear actuator model TNA08A25-S (Zaber Technologies Inc., Canada) used in combination with the MAX-232 (RS, Electrocomponents plc, UK) was used to form a syringe pump. The actuator has a speed resolution of 0.22 μm/s (flow rate accuracy using cartridges modified from 3 ml BD syringes: 48 µl/h) and thrust force of 50 N. A PIC16 microcontroller (Microchip Technology Inc., US) and a LCD 8 × 2 character display (RS, Electrocomponents plc, UK) were used to provide user interface for the operator to control the device. A pack of 10 rechargeable NiMH AA batteries (RS, Electrocomponents plc, UK) was assembled to supply sufficient power to the device to function for approx. 2 h. Generic electronic components were used to complete the circuit board and to allow user input generic switches and push buttons were used. Software, electrical components and the SLS (selective laser sintering) printed casing were assembled at Triteq Ltd (UK). The pen extension used for manual spinning was made of a 40 cm long Teflon tube (Sigma-Aldrich) ending with a pen-shaped casing. The tube was secured in the pen with appropriate fittings.

### Preparation of polymer solutions

Poly(ε-caprolactone) (PCL, Mw 80 kDa, Sigma-Aldrich) was dissolved into 1,1,1,3,3,3- hexafluoroisopropanol (HFIP, Apollo Scientific Ltd., Cheshire, UK) at 8 % (w/v). Poly(p-dioxanone) (PDO, viscosity 1.5-2.2 dl/g, Sigma-Aldrich) was dissolved into HFIP at 9 % (w/v). Poly(lactic-co-glycolic acid) (PLGA 75:25, Mw 66-107 kD, Sigma-Aldrich) was added in HFIP at 17 % (w/v) and 2.5 % (w/v). Poly(3-hydroxybutyrate) (PHB, Mw 6kD, Polysciences Europe GmbH, Eppelheim, Germany) was dissolved at 4.5 % (w/v) in HFIP. Poly(vinyl acohol) (PVA, Mw 89-98 kD, 99 % hydrolysed, Sigma-Aldrich) was dissolved at 10 % (w/v) in deionised water at 90 °C for at least 6 h under constant stirring. Poly(ethylene oxide) (PEO, Mw 400 kD, Sigma-Aldrich) was added to deionised water at 4 % (w/v). Poly(vinyl butyral) (PVB, Mowital B75H, Kuraray Europe GmbH, Hattersheim, Germany) was dissolved at 8 % (w/v) in ethanol/methanol (9:1 v/v). Alginate (sodium alginate, 100–300 cP, Sigma-Aldrich) was dissolved in deionised water at 2 % (w/v) at 90 °C for 3 h with gentle stirring. All solutions were agitated at room temperature on a roller for at least 24 h prior electrospinning or electrospraying.

### Electrospinning

Polymer solutions were loaded into 2 ml cartridges. Cartridges were produced by shortening both the main body and the plunger of 3 ml syringes with a Luer lock (BD, Oxford, UK). The syringes were fitted with blunted needles, gauge 21. Electrospinning with the portable electrospinner was performed horizontally with a distance of 20 cm and a flow rate of 1 ml/h. The voltage was adjusted with each polymer solution to obtain a stable jet. Fibres were collected onto a stainless steel plate covered with aluminium foil. The plate was grounded to the apparatus via a cable. Experiments involving the use of HFIP solvent were carried out within a glove box purged with nitrogen. Electrospinning on skin was performed with the pen extension, manually maintained at constant distance by the experimenter during the spinning. The ground cable was kept in contact with the skin. Demonstrations performed with pig skin samples were carried out with the PDO solution. Demonstrations performed on human volunteers were carried out with the PEO solution. Benchtop electrospinning was performed using an apparatus described by Mouthuy et al. (2010).

### Electrospraying

The alginate solution was electrosprayed at 2 ml/h into a grounded 0.102 M CaCl2 bath 6 cm underneath the tip of the extension. The 2.5 % PLGA solution was electrosprayed at 20 cm onto a flat stainless steel collector covered with aluminium foil with a flow rate of 0.6 ml/h.

### Scanning electron microscopy (SEM)

Samples were mounted on an aluminium stub using a carbon adhesive disk and gold coated using a SC7620 Mini Sputter Coater System (Quorum Technologies Ltd, East Sussex). High resolution images were taken using an environmental SEM (Carl Zeiss Evo LS15 Variable Pressure SEM). Fibre diameters were measured using ImageJ software (by W. S. Rasband, US National Institutes of Health, Bethesda, Maryland, USA).

## Results and discussion

### Electrospinner design

**Fig. 1:**
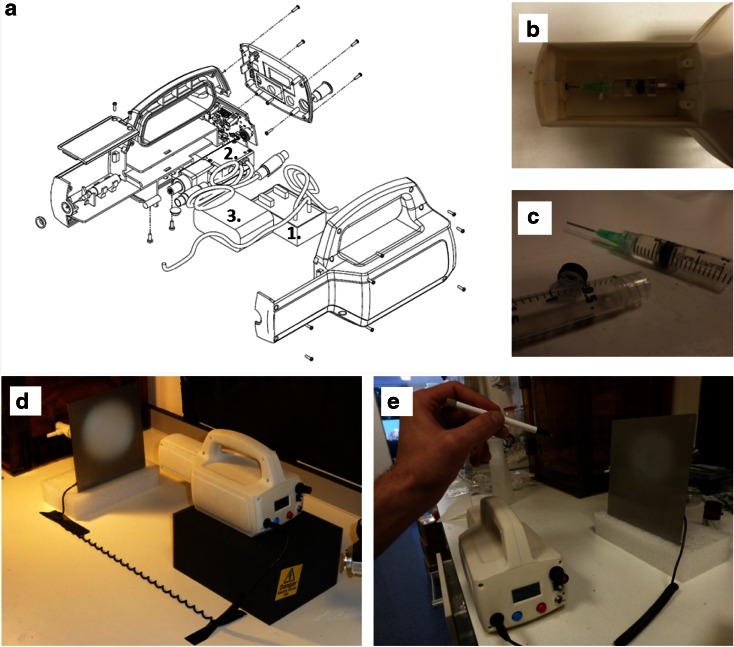
Design of the portable electrospinning apparatus. **a** Exploded view of the CAD drawing assembly of the device (1. high voltage converter, 2. linear actuator, 3. battery pack). **b** Separated compartment for cartridges, accessible from removable lid. **c** Cartridges used in this study. **d** Fibres being electrospun directly from the device. **e** Fibres being electrospun manually via a pen extension connected to the device

The portable electrospinner is displayed in Fig. 1. The high voltage converter, the linear actuator and the batteries are efficiently packed in the casing to minimise the size of the apparatus (Fig. 1a). As shown in Fig. 1b, cartridges are located in a separate compartment to allow safe and easy replacement. The modified syringes used as cartridges are seen in Fig. 1c. The total weight of the apparatus is 1.11 kg. The electrospinning can be used by spinning directly from the cartridge (Fig. 1d) or with the pen extension (Fig. 1e). The voltages can be selected between 0 and 14 kV and a wide choice of flow rates were pre-set between 0 and 10 ml/h. Values are indicated on the LCD screen during use. Batteries were able to provide enough power for the device to run for about 105 min at 13 kV and 1 ml/h.

### Electrospinning performances

**Fig. 2:**
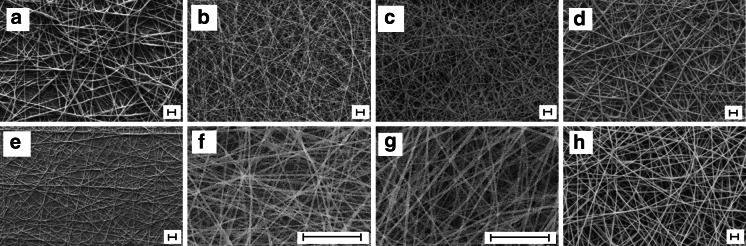
Fibres electrospun with the portable device from different polymer solutions: **a** PCL 8 % in HFIP, **b** PCL 8 % in HFIP, **c** PDO 9 % in HFIP, **d** PLGA 17 % in HFIP, **e** PHB 8 % in HFIP, **f** PEO 4 % in H2O, **g** PVA 10 % in H2O, **h** PVB 4.5 % in Ethanol/methanol. *Scale bars* represent 10 μm

**Fig. 3:**
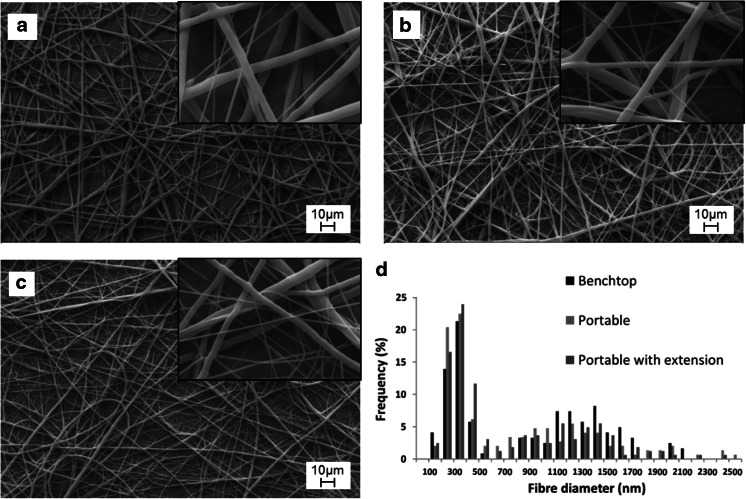
Morphologies of PCL scaffolds produced with benchtop and portable electrospinning devices. **a** Mesh electrospun with benchtop electrospinning apparatus. **b** Mesh electrospun with the portable apparatus. **c** Mesh electrospun with the portable apparatus and the pen extension. **d** Frequency of the fibre diameters observed with each electrospinning setup. Experiments were performed with PCL 8 % in HFIP and identical electrospinning parameters with each setup

As seen in Fig. 2, the electrospinner is able to spin fibres made of various polymers including PCL, PDO, PLGA, PHB, PEO, PVA and PVB. Most of these materials are commonly used in electrospinning research and the range includes both water-soluble and non-water-soluble polymers. Electrospinning parameters and fibres diameters are indicated in Table 1. The average diameters varied between 268 nm and 1,607 nm depending on the solutions and parameters used. This demonstrates the ability of the device to produce both sub- and sup-micron fibres. In Fig. 3, we compare the morphologies of PCL electrospun fibres produced with the portable device (with and without extension) to those produced with a benchtop device. SEM images (Fig. 3a–c) show that similar structures were obtained. Moreover, as shown in Fig. 3d, the fibre diameter distribution profiles are almost identical, regardless the electrospinning apparatus used. In all three configurations (benchtop, portable and portable with extension), PCL scaffolds display two peaks of diameters around 350 nm and 1,300 nm. This is reflected in the high magnification SEM images where both sub- and sup-micron fibres are observed. Parameters and fibres diameters are indicated in Table 2. Although the portable device currently offers lower resolutions for both the voltage and the flow rate, no significant change of the average fibre diameter is observed. These results demonstrate the equivalence between a portable apparatus and a traditional benchtop setup since both produce similar scaffolds using identical process parameters.

**Table 1:** Electrospinning parameters used for each polymer solution and average fibre diameters observed in the resulting scaffolds

Polymer	Solvent	Concentration (%w/v)	Voltage (kV)	F (ml/h)	Distance (cm)	Fibre diameter (nm)
PCL	HFIP	8	8.1	1	20	786 ± 592
PCL	HFIP	8	11	1	20	566 ± 258
PDO	HFIP	9	10	1	20	982 ± 194
PLGA	HFIP	17	9.6	1	20	1,405 ± 626
PHB	HFIP	4.5	10.6	1	20	853 ± 273
PEO	H_2_O	4	10	1	20	268 ± 74
PVA	H_2_O	10	13.5	1	20	310 ± 96
PVB	Ethanol/methanol (9:1, v/v)	8	13.1	1	20	1,607 ± 207

**Table 2:** Electrospinning parameters used for the comparison of performances between a benchtop apparatus and the portable device

Apparatus	Polymer solution (w/v)	Voltage (kV)	F (ml/h)	Distance (cm)	Fibre diameter (nm)
Benchtop	PCL 8 % in HFIP	8.1 ± 0.1	1 ± 0.003	20	889 ± 577
Portable	PCL 8 % in HFIP	8.1 ± 0.2	1 ± 0.048	20	786 ± 592
Portable, with extension	PCL 8 % in HFIP	8.1 ± 0.2	1 ± 0.048	20	747 ± 535

### Electrospraying performances

**Fig. 4:**
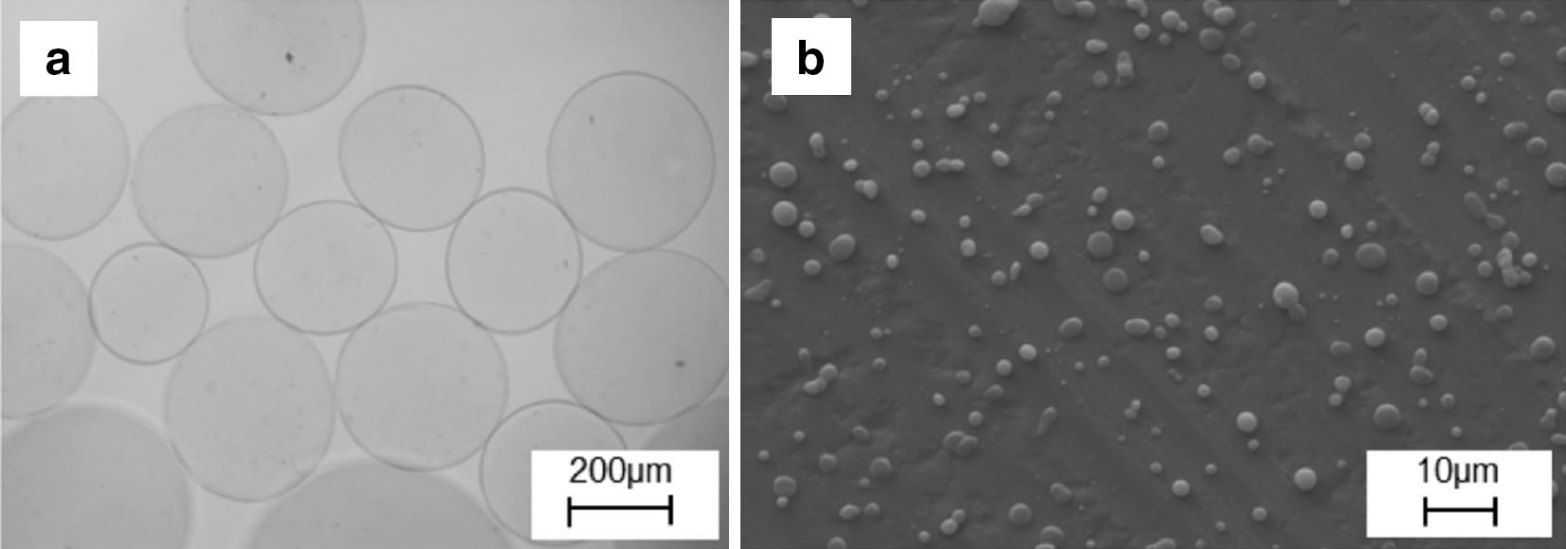
Morphologies of alginate beads and PLGA microparticles produced with the portable devices. **a** Beads produced from a 2 % alginate solution electrosprayed into a grounded CaCl2 bath. **b** Particles sprayed from a solution of 2.5 % PLGA in HFIP

The ability of the portable apparatus to electrospray beads and particles is shown in Fig. 4. Electrospraying parameters and measured particle diameters are listed in Table 3. These results confirm the suitability of the portable apparatus to be used as an electrospraying device. In particular, this suggests the potential of using the device for cell electrospraying applications (Jayasinghe et al. 2006).

**Table 3:** Electrospraying parameters used for the alginate and PLGA solutions

Polymer	Solvent	Concentration (%w/v)	Voltage (kV)	F (ml/h)	Distance (cm)	Particle diameter (μm)
Alginate	H_2_O	2	7.1	2	6	369 ± 105
PLGA	HFIP	2.5	11.2	0.6	20	1.9 ± 0.7

### In situ electrospinning

**Fig. 5:**
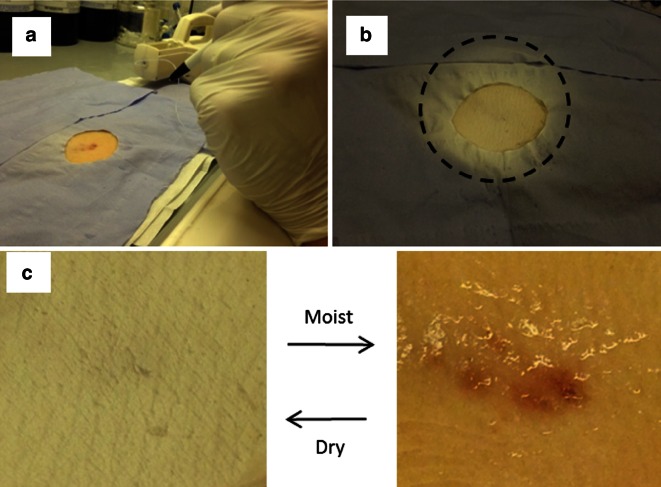
In situ electrospinning on pig skin. **a** PDO electrospun fibres being spun directly on a skin defect with the portable tool and the pen extension. **b** Skin defect fully covered by an electrospun patch (*dashed circle* electrospun fibres deposition area). **c** The skin defect can clearly be seen by moisturising the mesh. After drying, the patch recovers its opacity

**Fig. 6:**
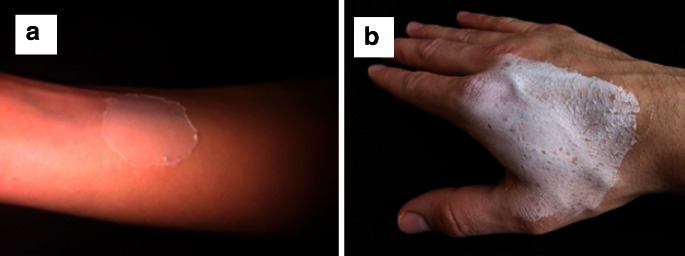
Examples of PEO patches created on the **a** arm and **b** hand of healthy volunteers

Results of in situ electrospinning experiments are presented in Figs. 5 and 6. Electrospinning parameters are displayed in Table 4. Fibres were successfully spun on both pig and human skins. The duration of electrospinning was selected to make the area of collection easily observable although a few minutes of electrospinning are sufficient to deposit several layers of electrospun fibres. Faster deposition rates could be achieved using a multi-nozzle setup or with a modified extension allowing multi-jet electrospinning. Figure 5a shows electrospun fibres being spun directly on a skin defect with the portable tool and the pen extension. The area of deposition of the fibres was reduced by the use of a mask, as shown in Fig. 5b. The electrospun mesh becomes completely transparent after being moisturised (Fig. 5c), allowing clear visualisation of the skin defect through the fibres. After drying, the mesh recovers its opacity. This interesting property may be used to monitorthe healing of a wound without removing a deposited electrospun patch.

**Table 4:** Solutions and parameters used for the electrospinning experiments carried out on skin

Targeted skin	Polymer solution (w/v)	Voltage (kV)	F (ml/h)	Distance (cm)	Duration (min)
Pig	PDO 9 % in HFIP	10	1	20	30
Human	PEO 4 % in H_2_O	10	1	20	5–15

### Future directions of portable electrospinning tools

The emergence of portable electrospinning technologies benefits from the progressive miniaturisation of batteries, high voltage converters and linear actuators. Eventually, it may lead to the design of pen-size electrodispersers. In the short term, these technologies are likely to be of benefit to academics as well as to industrial users. Table 5 lists the advantages and inconveniences of a portable tool compared to a benchtop device. The character of portability makes it possible to use the equipment in various places including laboratory benches, fume hoods (for organic solvent), cell culture hoods (for cell electrospinning/spraying), clean rooms (for implant preparation), classrooms (for demonstration purposes), and even outdoors. Since it allows more flexibility in their use such as in terms of direction of spinning and target, portable devices may lead to the discovery of new electrospinning applications. In particular, it may become simpler to explore the combination of electrospinning with other existing technologies such as with 3D printing. Another major advantage of the portable device is their affordability, since all electronic components involved are relatively inexpensive. In the long term, portable electrospinning technologies are likely to help for wound healing management and other conditions that may require controlled release of bioactive components. They could be particularly useful to regenerative medicine approaches using electrospinning and electrospraying of living cells. However, there is still work to be done to demonstrate the real benefit of in situ electrospinning and to ensure that the apparatus complies with safety regulations.

**Table 5:** Advantages and inconveniences of the portable device compared to a standard benchtop apparatus

Benchtop	Portable
Advantages	
Control over ambient parameters	Transportability (small, light, handheld)
Higher voltages available	Flexibility in use (e.g. direction, target type)
Accuracy on voltage and flow rate	In situ spinning/spraying
Safety management	Manual spinning/spraying
	Battery powered
	Affordable
	Medical applications
Inconveniences	
Difficult to transport	Limited by performance of converter
Need for power source	Limited by capacity of batteries
Lack of flexibility	Fixed cartridge size
Expensive	

## Conclusions

A unique electrospinning apparatus, which is portable and battery-powered, is presented. Both the voltage and flow rate can be adjusted over a range of values suitable for most electrospinning applications via a microcontroller. A pen extension device can be used for manual spinning. The apparatus was able to electrospin sub- and sup-micron fibres made of several polymers commonly used in the field of electrospinning and the quality of the non-woven meshes was similar to those made with a benchtop device. Electrospraying beads and microparticles was also achieved. Finally, we shown the possibility of electrospinning directly onto skin. Overall, these results demonstrate that portable electrospinning devices could become an attractive alternative to existing benchtop machines in the near future. They offer advantages in terms of cost, transportability and flexibility of use which may be particularly beneficial to the development of new wound healing strategies.

## References

[CR1] Alves da Silva ML, Martins A, Costa-Pinto AR, Costa P, Faria S, Gomes M, Reis RL, Neves NM (2010). Cartilage tissue engineering using electrospun PCL nanofiber meshes and MSCs. Biomacromolecules.

[CR2] Aviss KJ, Gough JE, Downes S (2010). Aligned electrospun polymer fibres for skeletal muscle regeneration. Eur Cells Mater.

[CR3] Coffee RA (1998) A dispensing device and method for forming material. CA Patent App CA 2,296,334

[CR4] Downing TL, Soto J, Morez C, Houssin T, Fritz A, Yuan F, Chu J, Patel S, Schaffer DV, Li S (2013). Biophysical regulation of epigenetic state and cell reprogramming. Nat Mater.

[CR5] Greiner A, Wendorff JH (2007). Electrospinning: a fascinating method for the preparation of ultrathin fibers. Angew Chem Int Ed.

[CR6] Jayasinghe SN (2013). Cell electrospinning: a novel tool for functionalising fibres, scaffolds and membranes with living cells and other advanced materials for regenerative biology and medicine. Analyst.

[CR7] Jayasinghe SN, Qureshi AN, Eagles PAM (2006). Electrohydrodynamic jet processing: an advanced electric-field-driven jetting phenomenon for processing living cells. Small.

[CR8] Kumbar SG, Nukavarapu SP, James R, Nair LS, Laurencina CT (2008). Electrospun poly(lactic acid-co-glycolic acid) scaffolds for skin tissue engineering. Biomaterials.

[CR9] Liu YJ, Ramaseshan R, Dong YX, Kumar A, Ramakrishna S (2010) A portable electrospinning apparatus. WO Patent App PCT/SG2008/000,444

[CR10] Liu S, Li K, Xu L, Long S, Liu Y, Sun B, Zhang H (2012) Portable handheld electrostatic spinning device. CN Patent App CN 201,210,229,010

[CR11] Mouthuy PA, Ye H, Triffitt J, Oommen G, Cui Z (2010). Physico-chemical characterisation of functional electrospun scaffolds for bone and cartilage tissue engineering. Proc Inst Mech Eng H.

[CR12] Pirrie AB, Coffee RA (2003) Dispensing device. US Patent 6,595,208

[CR13] Reneker DH, Yarin AL (2008). Electrospinning jets and polymer nanofibers. Polymer.

[CR14] Rho KS, Jeong L, Lee G, Seo BM, Park YJ, Hong SD, Roh S, Cho JJ, Park WH, Min BM (2006). Electrospinning of collagen nanofibers: effects on the behavior of normal human keratinocytes and early-stage wound healing. Biomaterials.

[CR15] Sahoo S, Ouyang H, Goh JCH, Tay TE, Toh SL (2006). Characterization of a novel polymeric scaffold for potential application in tendon/ligament tissue engineering. Tissue Eng.

[CR16] Smith DJ, Reneker DH, McManus AT, Schreuder-Gibson HL, Mello C, Sennett MS (2004) Electrospun fibers and an apparatus therefor. US Patent 6,753,454

[CR17] Townsend-Nicholson A, Jayasinghe SN (2006). Cell electrospinning: a unique biotechnique for encapsulating living organisms for generating active biological microthreads/scaffolds. Biomacromolecules.

[CR18] Yang Y, Xia T, Chen F, Wei W, Liu C, He S, Li X (2012). Electrospun fibers with plasmid BFGF polyplex loadings promote skin wound healing in diabetic rats. Mol Pharmacol.

[CR19] Zamani M, Prabhakaran MP, Ramakrishna S (2013). Advances in drug delivery via electrospun and electrosprayed nanomaterials. Int J Nanomedicine.

[CR20] Zonari A, Novikoff S, Electo NRP, Breyner NM, Gomes DA, Martins A, Neves NM, Reis RL, Goes AM (2012). Endothelial differentiation of human stem cells seeded onto electrospun polyhydroxybutyrate/polyhydroxybutyrate-cohydroxyvalerate fiber mesh. PLoS One.

[CR21] Zong X, Li S, Chen E, Garlick B, Kim K, Fang D, Chiu J, Zimmerman T, Brathwaite C, Hsiao BS, Chu B (2004). Prevention of postsurgery-induced abdominal adhesions by electrospun bioabsorbable nanofibrous poly(lactide-coglycolide)-based membranes. Ann Surg.

